# Supplementary material for: Comparative Exergy and Environmental Assessment of the Residual Biomass Gasification Routes for Hydrogen and Ammonia Production
Source: Entropy (Basel). 2023 Jul 22;25(7):1098. doi: 10.3390/e25071098 (PMC10378585; doi:10.3390/e25071098)
Supplement: Supplementary file 1 [file entropy-25-01098-s001.zip › entropy-2440225-supplementary.pdf]

Flow tables referring to the gasification unit

Table S1 - Streams for gasification unit: Sugarcane bagasse

Streams	1	2	3	4	5	6	7	8
				**Molar Fraction**				
CO	--	--	--	--	--	0.184	0.200	0.263
CO_2	--	--	--	--	0.161	0.119	0.130	0.170
H_2	--	--	--	--	--	0.347	0.378	0.496
H_2O	--	1	1	--	--	0.299	0.239	0.002
N_2	--	--	--	0.790	0.790	--	--	--
CH_4	--	--	--	--	--	0.049	0.053	0.069
NH_3	--	--	--	--	--	0.002	0.001	--
H_2S	--	--	--	--	--	--	--	--
O_2	--	--	--	0.210	0.049	--	--	--
				**Mass Fraction**				
Biomass	1	--	--	--	--	--	--	--
CO	--		--	--	--	0.298	0.326	0.434
CO_2	--	--	--	--	0.230	0.303	0.331	0.440
H_2	--	--	--	--	--	0.040	0.044	0.059
H_2O	--	1	1	--	--	0.311	0.249	0.002
N_2	--	--	--	0.767	0.719	--	--	--
CH_4	--	--	--	--	--	0.045	0.049	0.065
NH_3	--	--	--	--	--	0.001	0.001	--
H_2S	--	--	--	--	--	--	--	--
O_2	--	--	--	0.2329	0.0508	--	--	--
$\dot{n}$ (kmol/h)	0	314.03	314.03	962.47	962.47	1030.49	947.78	721.92
$\dot{m}$ (kg/h)	26640	5657.42	5657.42	27767.68	29630.50	17832.13	16342.51	12272.90
T (°C)	25	25	121	650	100	400	66	35
P (bar)	1	2	2	1	1	1	1	35

Table S2 - Streams for gasification unit: Sewage sludge

Streams	1	2	3	4	5	6	7	8
				**Molar Fraction**				
CO	--	--	--	--	--	0.166	0.187	0.244
CO_2	--	--	--	--	0.188	0.087	0.098	0.128
H_2	--	--	--	--	--	0.359	0.404	0.527
H_2O	--	1.000	1.000	--	--	0.250	0.200	0.001
N_2	--	--		0.790	0.790	0.001	0.002	0.002
CH_4	--	--	--	--	--	0.066	0.075	0.098
NH_3	--	--	--	--	--	0.067	0.032	--
H_2S	--	--	--	--	--	0.003	0.004	--
O_2	--	--	--	0.210	0.022	--	--	--
				**Mass Fraction**				
Biomass	1	--	--	--	--	--	--	--
CO	--	--	--	--	--	0.29	0.33	0.45
CO_2	--	--	--	--	0.27	0.24	0.27	0.37
H_2	--	--	--	--	--	0.05	0.05	0.07
H_2O	--	1.00	1.00	--	--	0.28	0.23	0.00
N_2	--	--	--	0.77	0.71	0.00	0.00	0.00
CH_4	--	--	--	--	--	0.07	0.08	0.10
NH_3	--	--	--	--	--	0.07	0.03	--
H_2S	--	--	--	--	--	0.01	0.01	--
O_2	--	--	--	0.23	0.02	--	--	--
$\dot{n}$ (kmol/h)	--	331.51	331.51	1059.25	1059.25	1305.14	1158.55	887.27
$\dot{m}$ (kg/h)	26640	5972.17	5972.17	30559.84	32948.14	20965.32	18374.35	13450.24
T (°C)	25	25	121	650	100	400	62	35
P (bar)	1	2	2	1	1	1	1	35

Table S3 - Streams for gasification unit: Orange Bagasse

Streams	1	2	3	4	5	6	7	8
				**Molar Fraction**				
CO	--	--	--	--	--	0.241	0.259	0.314
CO_2	--	--	--	--	0.153	0.117	0.126	0.153
H_2	--	--	--	--	--	0.330	0.354	0.429
H_2O	--	1.000	1.000	--	--	0.214	0.169	0.002
N_2	--	--	--	0.790	0.790	--	--	--
CH_4	--	--	--	--	--	0.079	0.085	0.103
NH_3	--	--	--	--	--	0.018	0.008	--
H_2S	--	--	--	--	--	--	--	--
O_2	--	--	--	0.210	0.057	--	--	--
				**Mass Fraction**				
Biomass	1	--	--	--	--	--	--	--
CO	--	--	--	--	--	0.375	0.402	0.487
CO_2	--	--	--	--	0.219	0.287	0.307	0.372
H_2	--	--	--	--	--	0.037	0.039	0.048
H_2O	--	1.000	1.000	--	--	0.214	0.169	0.002
N_2	--	--	--	0.767	0.721	0.001	0.001	0.001
CH_4	--	--	--	--	--	0.070	0.075	0.091
NH_3	--	--	--	--	--	0.017	0.007	--
H_2S	--	--	--	--	--	--	--	--
O_2	--	--	--	0.233	0.060	--	--	--
$\dot{n}$ (kmol/h)	0	368.76	368.76	1647.61	1647.61	1559.42	1454.77	1199.80
$\dot{m}$ (kg/h)	26640	6643.31	6643.31	47534.25	50553.16	28126.23	26257.19	21674.77
T (°C)	25	25	121	650	100	400	59	35
P (bar)	1	2	2	1	1	1	1	35

Flow tables referring to the syngas treatment unit for hydrogen production

Table S4 - Streams for the syngas treatment unit for hydrogen production: Sugarcane bagasse

Streams	1	2	3	4
		Molar Fraction		
CO	0.1627	0.0268	0.0268	0.0017
CO_2	0.0857	0.2215	0.2215	0.2466
H_2	0.2490	0.3848	0.3848	0.4099
H_2O	0.4511	0.3153	0.3153	0.2902
N_2	0.0000	0.0000	0.0000	0.0000
CH_4	0.0515	0.0515	0.0515	0.0515
		Mass Fraction		
CO	0.2562	0.0422	0.0422	0.0027
CO_2	0.2120	0.5482	0.5482	0.6103
H_2	0.0282	0.0436	0.0436	0.0465
H_2O	0.4570	0.3194	0.3194	0.2940
N_2	0.0000	0.0000	0.0000	0.0000
CH_4	0.0465	0.0465	0.0465	0.0465
$\dot{n}$ (kmol/h)	1243.95	1243.95	1243.95	1243.95
$\dot{m}$ (kg/h)	22121.30	22121.30	22121.30	22121.30
T (°C)	280	418	180	204
P (bar)	35	35	35	35

Table S5 - Streams for syngas treatment unit for hydrogen production: Sewage Sludge

Streams	1	2	3	4
		Molar Fraction		
CO	0.1494	0.0289	0.0289	0.0024
CO_2	0.0783	0.1988	0.1988	0.2253
H_2	0.3234	0.4439	0.4439	0.4704
H_2O	0.3877	0.2672	0.2672	0.2407
N_2	0.0013	0.0013	0.0013	0.0013
CH_4	0.0599	0.0599	0.0599	0.0599
		Mass Fraction		
CO	0.2574	0.0498	0.0498	0.0041
CO_2	0.2118	0.5380	0.5380	0.6097
H_2	0.0401	0.0550	0.0550	0.0583
H_2O	0.4295	0.2960	0.2960	0.2666
N_2	0.0022	0.0022	0.0022	0.0022
CH_4	0.0591	0.0591	0.0591	0.0591
$\dot{n}$ (kmol/h)	1447.27	1447.27	1447.27	1447.27
$\dot{m}$ (kg/h)	23538.79	23538.79	23538.79	23538.79
T (°C)	280	405	180	209
P (bar)	35	35	35	35

Table S6 - Streams for syngas treatment unit for hydrogen production: Orange Bagasse

**Streams**	**1**	**2**	**3**	**4**
		**Molar Fraction**		
CO	0.1872	0.0367	0.0367	0.0047
CO_2	0.0911	0.2416	0.2416	0.2735
H_2	0.2557	0.4062	0.4062	0.4381
H_2O	0.4046	0.2541	0.2541	0.2221
N_2	0.0003	0.0003	0.0003	0.0003
CH_4	0.0612	0.0612	0.0612	0.0612
		**Mass Fraction**		
CO	0.2906	0.0570	0.0570	0.0073
CO_2	0.2221	0.5891	0.5891	0.6671
H_2	0.0286	0.0454	0.0454	0.0489
H_2O	0.4039	0.2537	0.2537	0.2218
N_2	0.0005	0.0005	0.0005	0.0005
CH_4	0.0544	0.0544	0.0544	0.0544
ṅ (kmol/h)	2011.80	2011.80	2011.80	2011.80
ṁ (kg/h)	36303.17	36303.17	36303.17	36303.17
T (°C)	260	414	200	234
P (bar)	35	35	35	35

Flow tables referring to the syngas treatment unit for ammonia production

Table S7 - Streams for syngas treatment unit for ammonia production - Sugarcane bagasse

**Streams**	**1**	**2**	**3**	**4**	**5**	**6**	**7**	**8**
				**Molar Fraction**				
CO	--	--	0.09	0.052	0.052	0.004	0.004	--
CO_2	--	--	0.047	0.107	0.107	0.155	0.155	0.159
H_2	--	--	0.138	0.223	0.223	0.271	0.271	0.275
H_2O	--	--	0.578	0.524	0.524	0.476	0.476	0.472
N_2	0.790	0.790	0.094	0.092	0.092	0.092	0.092	0.092
CH_4	--	--	0.028	0.002	0.002	0.002	0.002	0.002
O_2	0.21	0.21	0.025	--	--	--	--	--
				**Mass Fraction**				
CO	--	--	0.131	0.078	0.078	0.006	0.006	0.001
CO_2	--	--	0.109	0.253	0.253	0.365	0.365	0.374
H_2	--	--	0.014	0.024	0.024	0.029	0.029	0.03
H_2O	--	--	0.543	0.506	0.506	0.46	0.46	0.456
N_2	0.767	0.767	0.138	0.138	0.138	0.138	0.138	0.138
CH_4	--	--	0.024	0.002	0.002	0.002	0.002	0.002
O_2	0.233	0.233	0.042	--	--	--	--	--
ṅ (kmol/h)	270.00	270.00	2253.60	2314.80	2314.80	2314.80	2314.80	2314.80
ṁ (kg/h)	7745.59	7745.59	43198.20	43198.20	43198.20	43198.20	43198.20	43198.20
T (°C)	25	650	649.8	864.3	300	349.4	200	204
P (bar)	1	35	20	35	35	35	35	35

Table S8 - Streams for syngas treatment unit for ammonia production - Sewage sludge

Streams	1	2	3	4	5	6	7	8
					**Molar Fraction**			
CO	--	--	0.085	0.063	0.063	0.006	0.006	0.001
CO_2	--	--	0.045	0.093	0.093	0.149	0.149	0.155
H_2	--	--	0.184	0.262	0.262	0.318	0.318	0.324
H_2O	--	--	0.512	0.472	0.472	0.416	0.416	0.41
N_2	0.790	0.790	0.111	0.108	0.108	0.108	0.108	0.108
CH_4	--	--	0.034	0.003	0.003	0.003	0.003	0.003
O_2	0.21	0.21	0.029	--	--	--	--	--
					**Mass Fraction**			
CO	--	--	0.128	0.098	0.098	0.01	0.01	0.001
CO_2	--	--	0.106	0.228	0.228	0.366	0.366	0.38
H_2	--	--	0.02	0.029	0.029	0.036	0.036	0.036
H_2O	--	--	0.497	0.474	0.474	0.417	0.417	0.412
N_2	0.767	0.767	0.168	0.168	0.168	0.168	0.168	0.168
CH_4	--	--	0.029	0.002	0.002	0.002	0.002	0.002
O_2	0.233	0.233	0.051	--	--	--	--	--
$\dot{n}$ (kmol/h)	270.00	270.00	2253.60	2314.80	2314.80	2314.80	2314.80	2314.80
$\dot{m}$ (kg/h)	7745.59	7745.59	43198.20	43198.20	43198.20	43198.20	43198.20	43198.20
T (°C)	25	650	650	885	300	359	200	206
P (bar)	1	35	20	35	35	35	35	35

Table S9 - Streams for syngas treatment unit for ammonia production - Orange bagasse

Streams	1	2	3	4	5	6	7	8
					**Molar Fraction**			
CO	--	--	0.127	0.101	0.101	0.017	0.017	0.002
CO_2	--	--	0.062	0.116	0.116	0.2	0.2	0.215
H_2	--	--	0.173	0.266	0.266	0.35	0.35	0.365
H_2O	--	--	0.437	0.392	0.392	0.308	0.308	0.293
N_2	0.79	0.79	0.127	0.122	0.122	0.122	0.122	0.122
CH_4	--	--	0.041	0.004	0.004	0.004	0.004	0.004
O_2	0.21	0.21	0.034	--	--	--	--	--
					**Mass Fraction**			
CO	--	--	0.179	0.149	0.149	0.025	0.025	0.003
CO_2	--	--	0.137	0.268	0.268	0.463	0.463	0.498
H_2	--	--	0.018	0.028	0.028	0.037	0.037	0.039
H_2O	--	--	0.398	0.372	0.372	0.292	0.292	0.278
N_2	0.767	0.767	0.18	0.18	0.18	0.18	0.18	0.18
CH_4	--	--	0.034	0.003	0.003	0.003	0.003	0.003
O_2	0.233	0.233	0.054	--	--	--	--	--
$\dot{n}$ (kmol/h)	0.133	0.133	0.827	0.861	0.861	0.861	0.861	0.861
$\dot{m}$ (kg/h)	13766.32	13766.32	58860.94	58860.94	58860.94	58860.94	58860.94	58860.94
T (°C)	25	650	650	906	300	389	200	217
P (bar)	1	35	20	35	35	35	35	35

Flow tables referring to the syngas purification unit for ammonia production

Table S10 - Streams for syngas purification unit for ammonia production - Sugarcane bagasse

Streams	1	2	3	4
		**Molar Fraction**		
CO	0.0010	0.0010	--	--
CO_2	0.3000	0.0060	0.9780	--
H_2	0.5200	0.7410	0.0090	0.7338
H_2O	0.0020	--	0.0060	--
N_2	0.1730	0.2460	0.0060	0.2532
CH_4	0.0040	0.0050	0.0010	0.0130
		**Mass Fraction**		
CO	0.0010	0.0040	--	--
CO_2	0.6870	0.0320	0.9930	--
H_2	0.0550	0.1700	--	0.1685
H_2O	0.0020	--	0.0020	--
N_2	0.2530	0.7850	0.0040	0.8079
CH_4	0.0030	0.0090	--	0.0237
$\dot{n}$ (kmol/h)	1223.62	853.67	369.96	828.90
$\dot{m}$ (kg/h)	23529.75	7492.59	16037.57	7278.87
T (°C)	35	25	25	25
P (bar)	35	35	1	35

Table S11 - Streams for syngas purification unit for ammonia production - Sewage sludge

Streams	1	2	3	4
		**Molar Fraction**		
CO	0.0010	0.0010	--	--
CO_2	0.2620	0.0050	0.9730	--
H_2	0.5480	0.7410	0.0120	0.7344
H_2O	0.0020	--	0.0070	--
N_2	0.1830	0.2460	0.0080	0.2524
CH_4	0.0050	0.0060	0.0010	0.0132
		**Mass Fraction**		
CO	0.0020	0.0050	--	--
CO_2	0.6450	0.0270	0.9910	--
H_2	0.0620	0.1710	0.0010	0.1690
H_2O	0.0020	--	0.0030	--
N_2	0.2860	0.7860	0.0050	0.8068
CH_4	0.0040	0.0110	--	0.0242
$\dot{n}$ (kmol/h)	1552.90	1140.73	412.18	1110.93
$\dot{m}$ (kg/h)	27788.08	9987.97	17800.62	9734.12
T (°C)	35	25	35	25
P (bar)	35	35	1	35

Table S12 - Streams for syngas purification unit for ammonia production - Orange bagasse

Streams	1	2	3	4
		**Molar Fraction**		
CO	0.002	0.003	--	--
CO_2	0.303	0.006	0.978	--
H_2	0.516	0.739	0.009	0.730
H_2O	0.002	--	0.006	--
N_2	0.172	0.245	0.006	0.253
CH_4	0.005	0.007	0.001	0.017
		**Mass Fraction**		
CO	0.003	0.011	--	--
CO_2	0.689	0.029	0.993	--
H_2	0.054	0.169	--	0.166
H_2O	0.002	0.000	0.002	--
N_2	0.248	0.779	0.004	0.803
CH_4	0.004	0.013	--	0.030
$\dot{n}$ (kmol/h)	2196.85	1524.72	672.13	1474.10
$\dot{m}$ (kg/h)	42579.26	13439.50	29140.50	13030.00
T (°C)	35	25	35	25
P (bar)	35	35	1	35

Flow tables referring to the syngas purification unit for hydrogen production

Table S13 - Streams for syngas purification unit for hydrogen production - sugarcane bagasse

Streams	1	2	3
	**Molar Fraction**		
CO	0.0020	0.0040	--
CO_2	0.3470	0.0080	0.9770
H_2	0.5760	0.8820	0.0080
H_2O	0.0020	--	0.0050
N_2	--	--	--
CH_4	0.0720	0.1060	0.0100
	**Mass Fraction**		
CO	0.0040	0.0260	--
CO_2	0.8630	0.0900	0.9940
H_2	0.0660	0.4520	0.0000
H_2O	0.0020	--	0.0020
N_2	--	--	--
CH_4	0.0660	0.4320	0.0040
$\dot{n}$ (kmol/h)	885.60	576.00	309.60
$\dot{m}$ (kg/h)	15643.65	2265.36	13378.60
T (°C)	35	25	35
P (bar)	35	35	1

Table S14 - Streams for syngas purification unit for hydrogen production - sewage sludge

**Streams**	**1**	**2**	**3**
	**Molar Fraction**		
CO	0.0030	0.0040	--
CO_2	0.2960	0.0070	0.9700
H_2	0.6180	0.8790	0.0110
H_2O	0.0020	--	0.0060
N_2	0.0020	0.0020	--
CH_4	0.0790	0.1070	0.0130
	**Mass Fraction**		
CO	0.0060	0.0310	--
CO_2	0.8300	0.0820	0.9920
H_2	0.0790	0.4420	0.0010
H_2O	0.0020	--	0.0020
N_2	0.0030	0.0160	--
CH_4	0.0800	0.4280	0.0050
$\dot{n}$ (kmol/h)	1101.60	770.40	331.20
$\dot{m}$ (kg/h)	17296.37	3086.23	14210.50
T (°C)	35	25	35
P (bar)	35	35	1

Table S15 - Streams for syngas purification unit for hydrogen production - Orange bagasse

**Streams**	**1**	**2**	**3**
	**Molar Fraction**		
CO	0.0060	0.0090	--
CO_2	0.3510	0.0090	0.9770
H_2	0.5620	0.8650	0.0070
H_2O	0.0020	--	0.0050
N_2	--	0.0010	--
CH_4	0.0790	0.1160	0.0110
	**Mass Fraction**		
CO	0.0090	0.0600	--
CO_2	0.8560	0.0950	0.9930
H_2	0.0630	0.4070	--
H_2O	0.0020	--	0.0020
N_2	0.0010	0.0040	--
CH_4	0.0700	0.4330	0.0040
$\dot{n}$ (kmol/h)	1567.68	1013.95	553.73
$\dot{m}$ (kg/h)	28300.45	4343.35	23957.64
T (°C)	35	25	35
P (bar)	35	35	1

Flow tables referring to the hydrogen production unit

Table S16 - Streams for hydrogen production unit - Sugarcane Bagasse

Streams	1	2	3	4	5
			**Molar Fraction**		
CO	0.0037	--	0.0227	0.0001	--
CO_2	0.0080	--	0.0495	0.0650	--
H_2	0.8821	1.0000	0.2723	--	1.0000
H_2O	--	--	0.0002	0.1415	--
N_2	0.0001	--	0.0003	0.7298	--
CH_4	0.1061	--	0.6550	--	--
O_2	0.0000	--	--	0.0637	--
			**Mass Fraction**		
CO	0.0262	--	0.0459	0.0001	--
CO_2	0.0895	--	0.1568	0.1025	--
H_2	0.4516	1.0000	0.0395	--	1.0000
H_2O	0.0002	--	0.0003	0.0914	--
N_2	0.0004	--	0.0007	0.7330	--
CH_4	0.4322	--	0.7569	--	--
O_2	--	--	--	0.0731	--
$\dot{n}$ (kmol/h)	575.25	482.07	93.18	1041.88	482.07
$\dot{m}$ (kg/h)	2265.36	971.81	1293.55	29059.61	971.81
T (°C)	26	26	26	100	35
P (bar)	35	35	1	1	200

Table S17 - Streams for hydrogen production unit - Sewage Sludge

Streams	1	2	3	4	5
			**Molar Fraction**		
CO	0.0044	--	0.0267	0.0001	--
CO_2	0.0075	--	0.0452	0.0650	--
H_2	0.8789	1.0000	0.2662	--	1.0000
H_2O	--	--	0.0002	0.1412	--
N_2	0.0024	--	0.0142	0.7303	--
CH_4	0.1069	--	0.6475	--	--
O_2	--	--	--	0.0634	--
			**Mass Fraction**		
CO	0.0308	--	0.0531	0.0001	--
CO_2	0.0820	--	0.1415	0.1026	--
H_2	0.4424	1.0000	0.0382	--	1.0000
H_2O	0.0002	--	0.0003	0.0912	--
N_2	0.0164	--	0.0284	0.7334	--
CH_4	0.4282	--	0.7386	--	--
O_2	--	--	--	0.0727	--
$\dot{n}$ (kmol/h)	770.65	643.42	127.22	1406.39	643.42
$\dot{m}$ (kg/h)	3086.30	1297.07	1789.23	39229.81	1297.07
T (°C)	26	26	26	100	35
P (bar)	35	35	1	1	200

Table S18 - Streams for hydrogen production unit - Orange bagasse

Streams	1	2	3	4	5
**Molar Fraction**					
CO	0.0092	--	0.0519	0.0001	--
CO_2	0.0093	--	0.0521	0.0684	--
H_2	0.8652	1.0000	0.2430	--	1.0000
H_2O	--	--	0.0002	0.1402	--
N_2	0.0006	--	0.0032	0.7290	--
CH_4	0.1156	--	0.6495	--	--
O_2	--	--	--	0.0622	--
**Mass Fraction**					
CO	0.0605	--	0.0986	0.0001	--
CO_2	0.0953	--	0.1555	0.1077	--
H_2	0.4072	1.0000	0.0332	--	1.0000
H_2O	0.0001	--	0.0002	0.0904	--
N_2	0.0038	--	0.0061	0.7306	--
CH_4	0.4331	--	0.7063	--	--
O_2	--	--	--	0.0713	--
ṅ (kmol/h)	1013.95	833.43	180.53	1985.79	833.43
ṁ (kg/h)	4343.34	1680.09	2663.26	55511.77	1680.09
T (°C)	26	26	26	100	35
P (bar)	35	35	1	1	200

Flow tables referring to the ammonia production unit

Table S19 - Streams for ammonia production unit - Sugarcane Bagasse

Streams	1	2	3	4	5	6	7	8	9
**Molar Fraction**									
H_2	0.6088	0.5126	0.5126	0.4691	0.4691	0.4372	0.0138	0.0059	0.4953
N_2	0.2842	0.2609	0.2609	0.2504	0.2504	0.2427	0.0136	0.0058	0.2741
CH_4	0.0832	0.0922	0.0922	0.0962	0.0962	0.0992	0.0195	0.0099	0.1102
NH_3	0.0238	0.1343	0.1343	0.1843	0.1843	0.2209	0.9531	0.9784	0.1204
**Mass Fraction**									
H_2	0.1123	0.0853	0.0853	0.0748	0.0748	0.0676	0.0016	0.0007	0.0799
N_2	0.7285	0.6037	0.6037	0.5548	0.5548	0.5216	0.0224	0.0096	0.6145
CH_4	0.1221	0.1221	0.1221	0.1221	0.1221	0.1221	0.0185	0.0093	0.1414
NH_3	0.0370	0.1888	0.1888	0.2482	0.2482	0.2887	0.9575	0.9804	0.1641
ṅ (kmol/h)	2411.15	2176.25	2176.25	2084.38	2084.38	2021.80	244.01	155.28	40.24
ṁ (kg/h)	26350.55	26350.55	26350.55	26350.55	26350.55	26350.55	4136.78	2639.17	502.75
T (°C)	350	511	404	469	400	444	30	-20	30
P (bar)	200	200	200	200	200	200	200	200	200

Table S20 - Streams for ammonia production unit - Sewage sludge

Streams	1	2	3	4	5	6	7	8	9
					**Molar Fraction**				
H_2	0.6243	0.5291	0.5291	0.4860	0.4860	0.4538	0.0142	0.0060	0.5163
N_2	0.2713	0.2464	0.2464	0.2352	0.2352	0.2268	0.0127	0.0056	0.2572
CH_4	0.0811	0.0899	0.0899	0.0939	0.0939	0.0969	0.0191	0.0097	0.1079
NH_3	0.0233	0.1346	0.1346	0.1850	0.1850	0.2225	0.9540	0.9788	0.1185
					**Mass Fraction**				
H_2	0.1192	0.0911	0.0911	0.0801	0.0801	0.0725	0.0017	0.0007	0.0868
N_2	0.7199	0.5898	0.5898	0.5389	0.5389	0.5037	0.0210	0.0092	0.6007
CH_4	0.1232	0.1232	0.1232	0.1232	0.1232	0.1232	0.0181	0.0091	0.1443
NH_3	0.0376	0.1959	0.1959	0.2577	0.2577	0.3005	0.9592	0.9810	0.1683
$\dot{n}$ (kmol/h)	3188.35	2875.70	2875.70	2753.39	2753.39	2668.83	332.28	199.75	59.36
$\dot{m}$ (kg/h)	33657.76	33657.76	33657.76	33657.76	33657.76	33657.76	5627.89	3394.14	712.13
T (°C)	350	512	405	470	400	446	30	-20	30
P (bar)	200	200	200	200	200	200	200	200	200

Table S21 - Streams for ammonia production unit - Orange bagasse

Streams	1	2	3	4	5	6	7	8	9
					**Molar Fraction**				
H_2	0.6014	0.5051	0.5051	0.4613	0.4613	0.4303	0.0137	0.0059	0.4856
N_2	0.2795	0.2559	0.2559	0.2451	0.2451	0.2375	0.0134	0.0058	0.2672
CH_4	0.0950	0.1051	0.1051	0.1098	0.1098	0.1130	0.0223	0.0113	0.1251
NH_3	0.0242	0.1339	0.1339	0.1839	0.1839	0.2191	0.9506	0.9771	0.1221
					**Mass Fraction**				
H_2	0.1104	0.0838	0.0838	0.0733	0.0733	0.0664	0.0016	0.0007	0.0780
N_2	0.7133	0.5898	0.5898	0.5411	0.5411	0.5092	0.0221	0.0095	0.5965
CH_4	0.1388	0.1388	0.1388	0.1388	0.1388	0.1388	0.0211	0.0106	0.1599
NH_3	0.0375	0.1876	0.1876	0.2468	0.2468	0.2856	0.9551	0.9792	0.1657
$\dot{n}$ (kmol/h)	4257.22	3845.24	3845.24	3682.97	3682.97	3576.38	418.91	277.11	97.21
$\dot{m}$ (kg/h)	46729.30	46729.30	46729.30	46729.30	46729.30	46729.30	7100.25	4709.37	1220.10
T (°C)	350	508	402	466	400	442	30	-20	30
P (bar)	200	200	200	200	200	200	200	200	200
